# Supplementary material for: ATP-dependent hydroxylation of an unactivated primary carbon with water
Source: Nat Commun. 2020 Aug 6;11:3906. doi: 10.1038/s41467-020-17675-7 (PMC7411048; doi:10.1038/s41467-020-17675-7)
Supplement: Supplementary file 1 — Supplementary Information [file 41467_2020_17675_MOESM1_ESM.pdf]

Supplementary Information

ATP-dependent hydroxylation of an unactivated primary carbon with water

Christian Jacoby[1†], Sascha Ferlaino[2], Dominik Bezold[3], Henning Jessen[3], Michael Müller[2] & Matthias Boll[1*]

[1]Microbiology, Faculty of Biology, Albert-Ludwigs-Universität Freiburg, Schänzlestr. 1, 79104 Freiburg, Germany

[2]Institute of Pharmaceutical Sciences, Albert-Ludwigs-Universität Freiburg, Albertstrasse 25, 79104 Freiburg, Germany

[3]Institute of Organic Chemistry, Albert-Ludwigs-Universität Freiburg, Albertstrasse 21, 79104 Freiburg, Germany

Supplementary Tables 1-4

Supplementary Figures 1-16

Supplementary Tables

Supplementary Table 1. ESI-Q-TOF analysis of substrates/products as determined in *in-vitro* assays. CDO = cholest-1,4-dien-3-one; DDO = desmost-1,4-dien-3-one; SD = standard deviation. Source data are provided as a Source Data file.

**Compound**	**Formula**	**(*m/z*) calculated**	**(*m/z*) determined**	**SD (Da)**	**Retention time (min)**
25-OH-CDO	$C_{27}H_{42}O_2$	399.3263 $[M+H]^+$	399.3274 $[M+H]^+$	1.1	4.22
25-phospho-CDO	$C_{27}H_{43}O_5P$	479.2926 $[M+H–PO_4^{3-}]^+$	479.2926 $[M+H–PO_4^{3-}]^+$	0.0	3.20
		381.3157 $[M+H]^+$	381.3154 $[M+H]^+$	0.3	
DDO	$C_{27}H_{40}O$	381.3157 $[M+H]^+$	381.3161 $[M+H]^+$	0.4	6.11
26-OH-DDO	$C_{27}H_{40}O_2$	397.3107 $[M+H]^+$	397.3109 $[M+H]^+$	0.2	4.06
DDO-26-al	$C_{27}H_{38}O_2$	395.2950 $[M+H]^+$	395.2953 $[M+H]^+$	0.3	4.31
DDO-26-oic acid	$C_{27}H_{38}O_3$	411.2899 $[M+H]^+$	411.2902 $[M+H]^+$	0.3	3.59

Supplementary Table 2. ESI-Q-TOF analyses of protein bands obtained during the enrichment of $S26DH_1$. Source data are provided as a Source Data file.

Mass of excised SDS protein band	Gene product identified	Score	Sequence coverage	Mass deduced from amino acid sequence
113	WP_154715926 (α5)	12513	73%	113
55	WP_154715926 (α5)	1129	29%	113
43	WP_107220515	8948	66%	42
40	WP_154715927 (β5)	9286	84%	40
20	WP_154715928 (γ5)	5534	80%	20

Supplementary Table 3. TAT-signal sequences alignments of Mo-dependent Type II DMSO reductases. The S/T**RR**X**FL/I** motive conserved in the TAT-signal sequence highlighted.

	10	20	30	40	50
S. denitrificans, α1	----------	-------**MQI**	**SRRQFIVGSA**	------**VAAA**	**GLGLYSLRPK**
S. denitrificans, α2	----------	----**MQFMQL**	**TRRHFIMGSA**	------**ATVA**	**GLALYSLRPR**
S. denitrificans, α3	----------	-------**MQV**	**SRRHFIVGTA**	------**AVAA**	**GAGLYSLRPK**
S. denitrificans, α4	----------	-------**MQV**	**SRRNFIVGSA**	------**VAAA**	**GLGLYSLKPK**
S. denitrificans, α5	------**MERS**	**SASSTVGLSV**	**SRRQFLIKAG**	---**LASMAGG**	**TLALFGCHRA**
S. denitrificans, α6	--------**MG**	**ILATSNLVSA**	**SRRKFLVMAG**	---**MASAAGA**	**AVGLFGCSRA**
S. denitrificans, α7	---------**M**	**TTASPAQPNP**	**ARRRFLILAG**	**KTTVAGIAAA**	**ATGLPGCNRM**
S. denitrificans, α8	----------	**MESKPGMIGM**	**DRRSFLKAGG**	------**SALA**	-**LSLCHLELL**
Strain HdN1, EbdA-like	-----**MTLGA**	**GMGILWKQKF**	**DRRSFLKASG**	---**YTVAAAA**	**AVELP**-----
Sdo. denitrificans, S25dA-like	--------**MF**	**KKDFRGGHMA**	**TRRHFLQAGV**	------**AVVG**	**SLPLRRLAFA**
A. aromaticum pCyN1, cmdA	----------	-----**MSVNF**	**DRRNFLKGSA**	------**ATVG**	**GLSLPSFIVE**
A. aromaticum, EbdA	**MDDLKNTDAI**	**RTGVSSAFDQ**	**NRRGFLKRSG**	------**AGAL**	**SLSLSSFAAG**
A. toluclasticus, EbdA	**MDERVKTGAA**	**ANGAS-TVDA**	**GRRNFLKGSG**	------**GLGL**	**SLSLPSFATA**
I. cernigliae, EbdA	-**MGKIDQGLS**	**MGTTEAGFSV**	**TRRGFLKGSG**	------**GAAL**	**ALSMSQLGGV**
A. aromaticum, EbdA 2	-**MTRDEMISV**	**EPEAAELQDQ**	**HRRDFLKRSG**	------**AAVL**	**SLSLSSLATG**
T. terpenica, EbdA-like	----------	-------**MTM**	**TRRSFIKNTG**	------**GALA**	-**LSLVQLQWK**
D. oleovorans Hxd3, EbdA-like	----------	----**MKEVKI**	**SRRTFLKGTS**	------**ATVA**	**LLSLNSLGFL**

Supplementary Table 4. Oligonucleotide primers used for heterologous production of DH_5, DH_6, DH_7, AcmB and C26-ALDH.

Primer	Sequence (5'-3')	T_a (°C)	Restriction site
DH_5_for	TGAGTCAAGCTTTGACCTAAGGAGGTAAATAATGG GCATACTCGCAAC	55	HindIII
DH_5_rev	ATGCTAACTAGTAATGACTCAAACCGCCG	55	SpeI
DH_6_for	TGAGTCAAGCTTTGACCTAAGGAGGTAAATAATGG AGCGGAGTTCAGC	57	HindIII
DH_6_rev	ATCGTAACTAGTAATTGCCGCGTCTCAAAC	57	SpeI
DH_7_for	ATCTGAAAGCTTTGACCTAAGGAGGTAAATAATGA CCACAGCCAGCC	58	HindIII
DH_7_rev	TATGTAACTAGTTGGGGATCAGGAATGAGCG	58	SpeI
SdhD_for	ATCGTAACTAGTTTAACTTTATAAGGAGGTGATAC GGTACATGCAAATGAGCAATGC	58	SpeI
SdhD_rev	TACGTATCTAGAATGAGCGATCAATGTGGCG	59	XbaI
AcmB_for	TGACTGGAGCTCTCAGGAAAGATAGAGGCGCGC	60	SacI
AcmB_rev	AGAGTCAAGCTTATGAGCATCGAAACCAACACATA TGAC	59	HindIII
C26-ALDH_for	TGAATCGCTAGCCATCATCACCATCACCACAACGA ACGAGGGCATG	55	NheI
C26-ALDH_rev	TGATACCCATGGTCAGGGTTTCACTGCCTTTTC	55	NcoI

Supplementary Figures

a

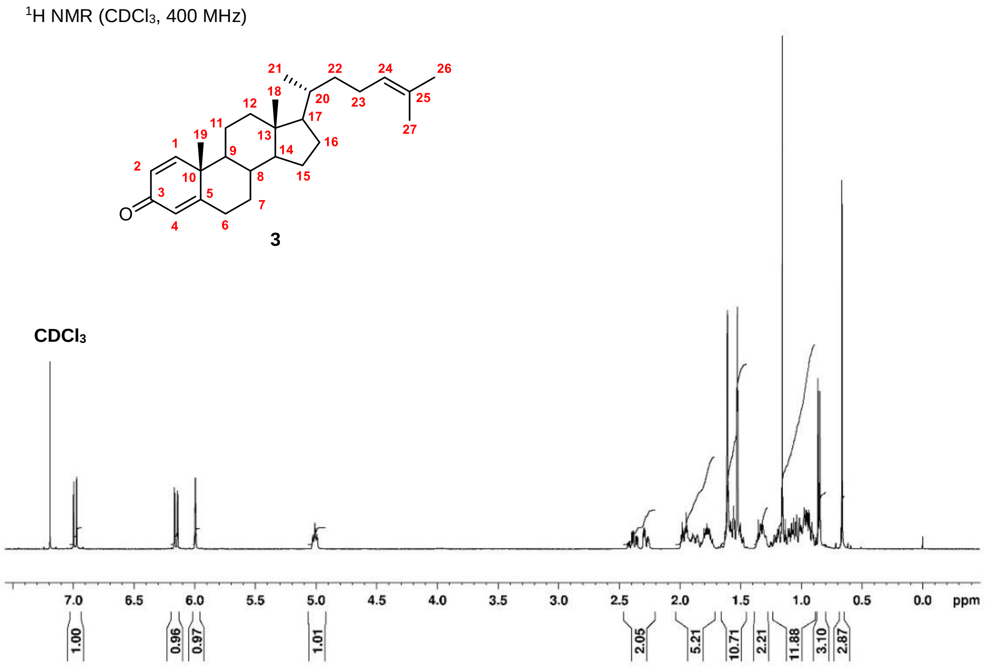

b

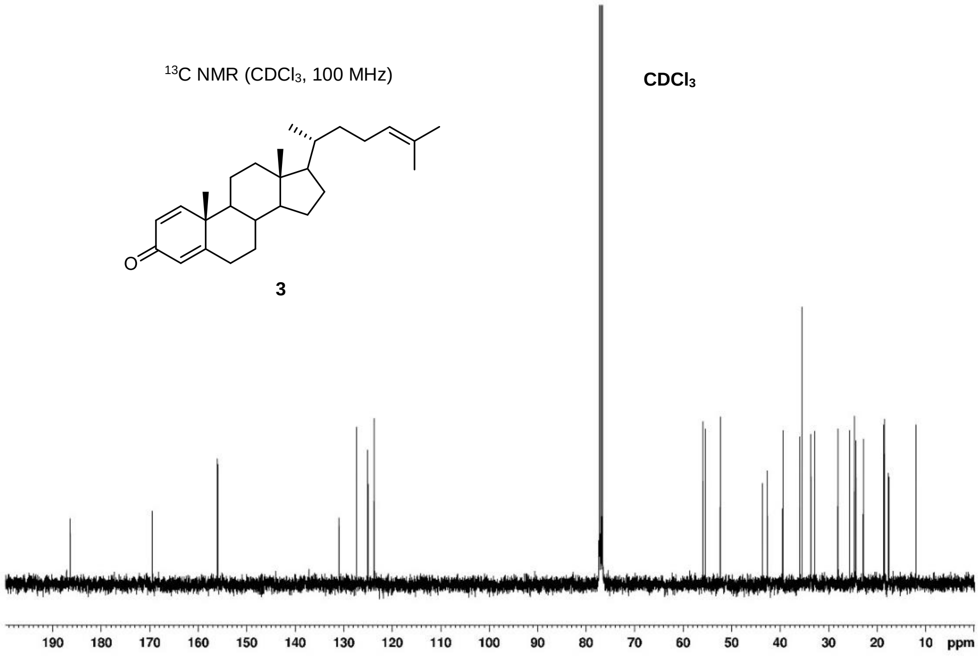

Supplementary Figure 1. ^{1}H (**a**) and ^{13}C (**b**) NMR spectra (400 MHz - ^{1}H, 100 MHz - ^{13}C, $CDCl_3$) of the product obtained during ATP-dependent conversion of 25-OH-CDO by *S. denitrificans* cell extracts. The spectra are indicative for desmost-1,4-diene-3-one (DDO, compound **3** in **Fig. 2**).

a

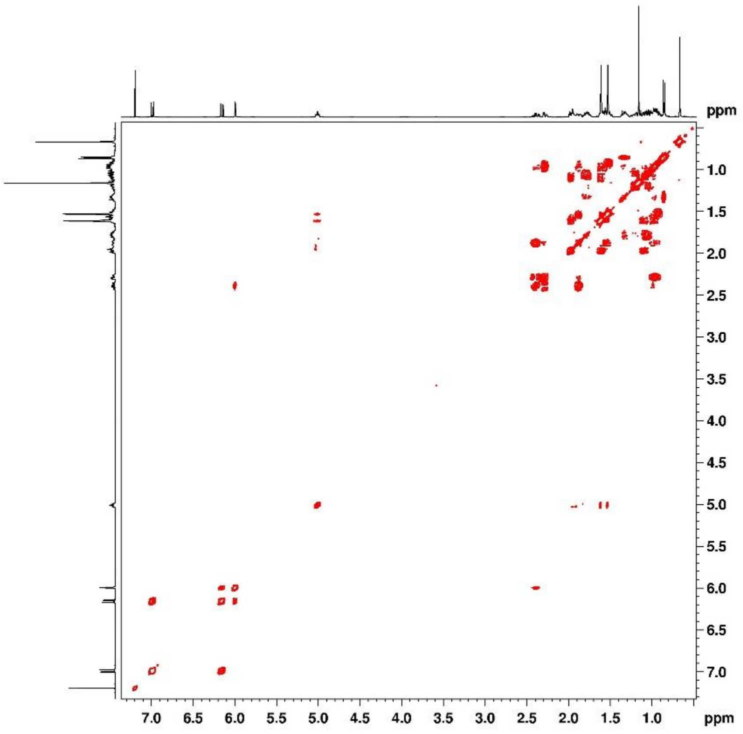

b

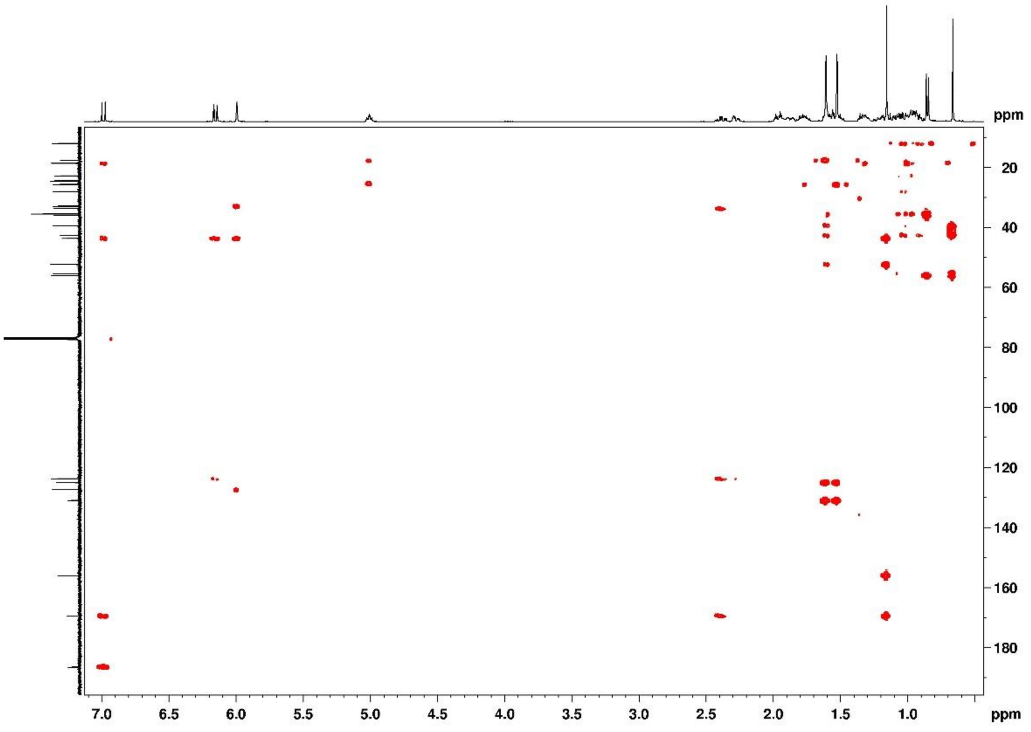

Supplementary Figure 2. HH COSY (**a**) and HMBC (**b**) NMR spectra of the product obtained during ATP-dependent conversion of 25-OH-CDO by *S. denitrificans* cell extracts. The spectra are indicative for desmost-1,4-diene-3-one (DDO, compound **3** in **Fig. 2**).

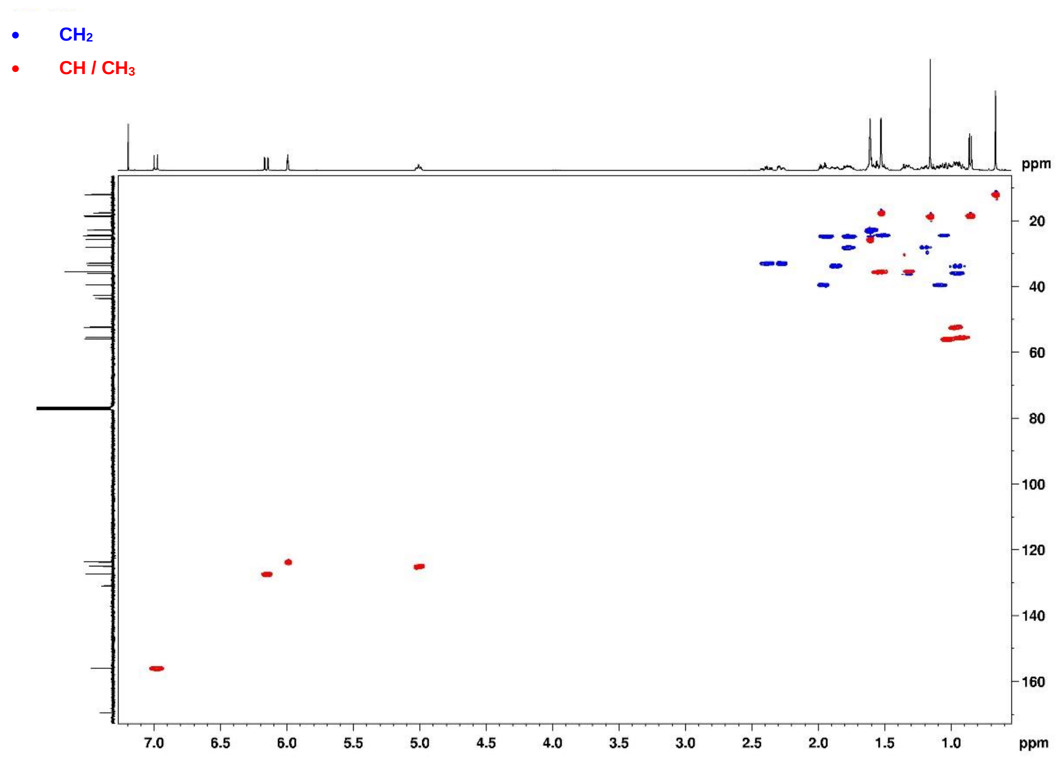

Supplementary Figure 3. HSQC NMR spectra of the product obtained during ATP-dependent conversion of 25-OH-CDO by *S. denitrificans* cell extracts. The spectra are indicative for desmost-1,4-diene-3-one (DDO, compound **3** in **Fig. 2**).

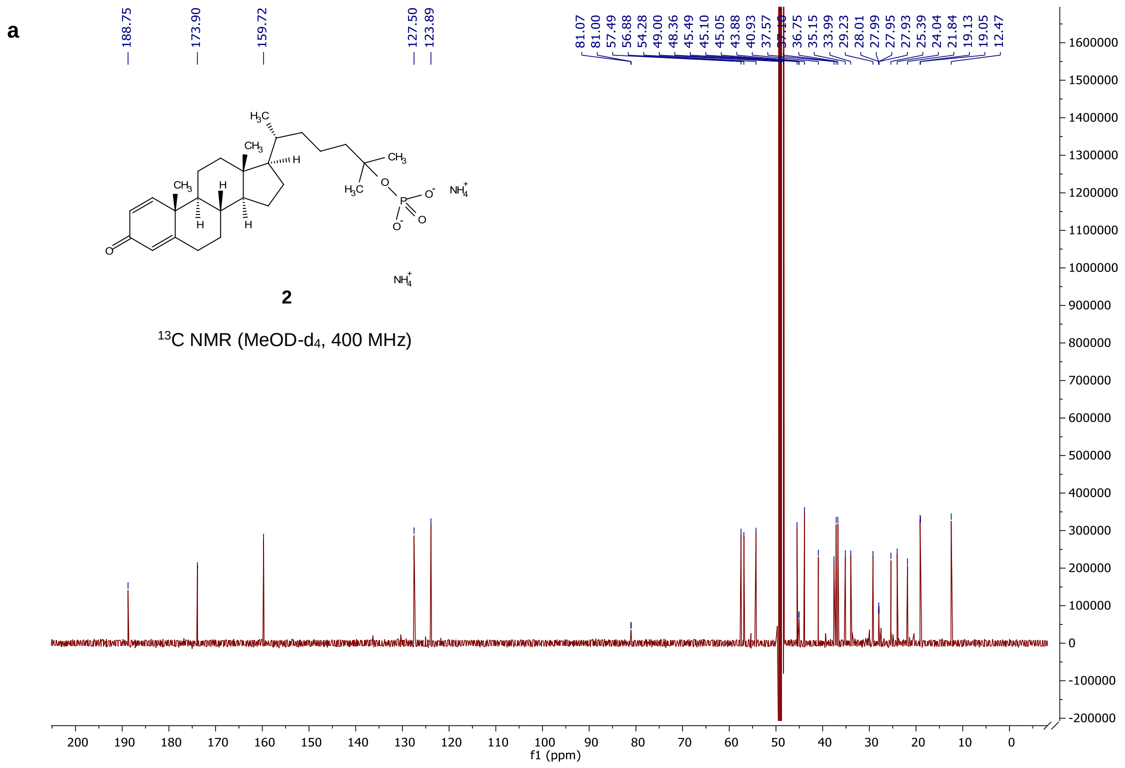

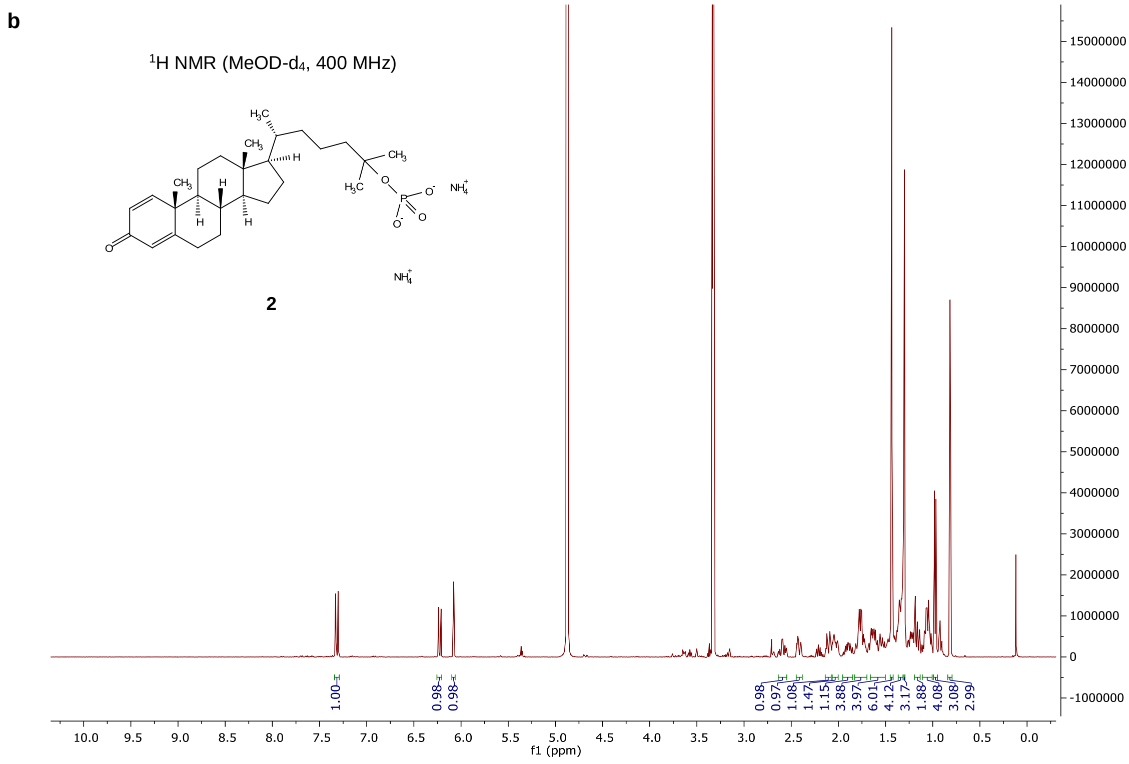

Supplementary Figure 4. ^{13}C (**a**) and ^{1}H (**b**) NMR-spectra (400 MHz, MeOD-d$_4$) of chemically synthesized 25-phospho-CDO, (**2**) in **Fig. 2**.

a

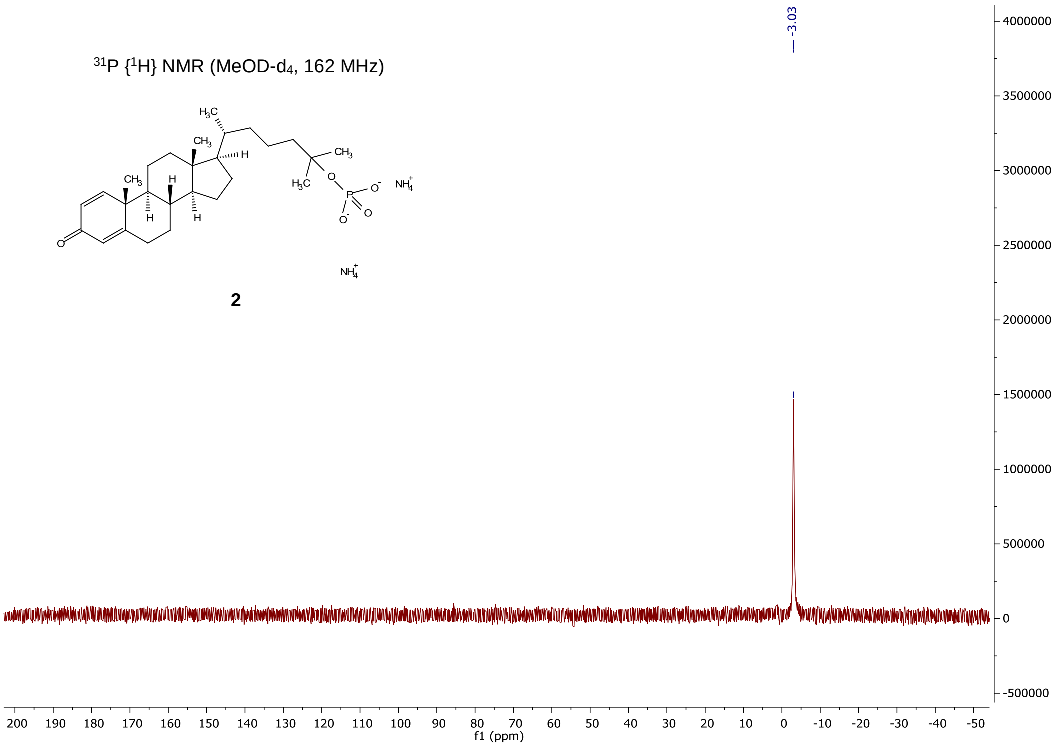

b

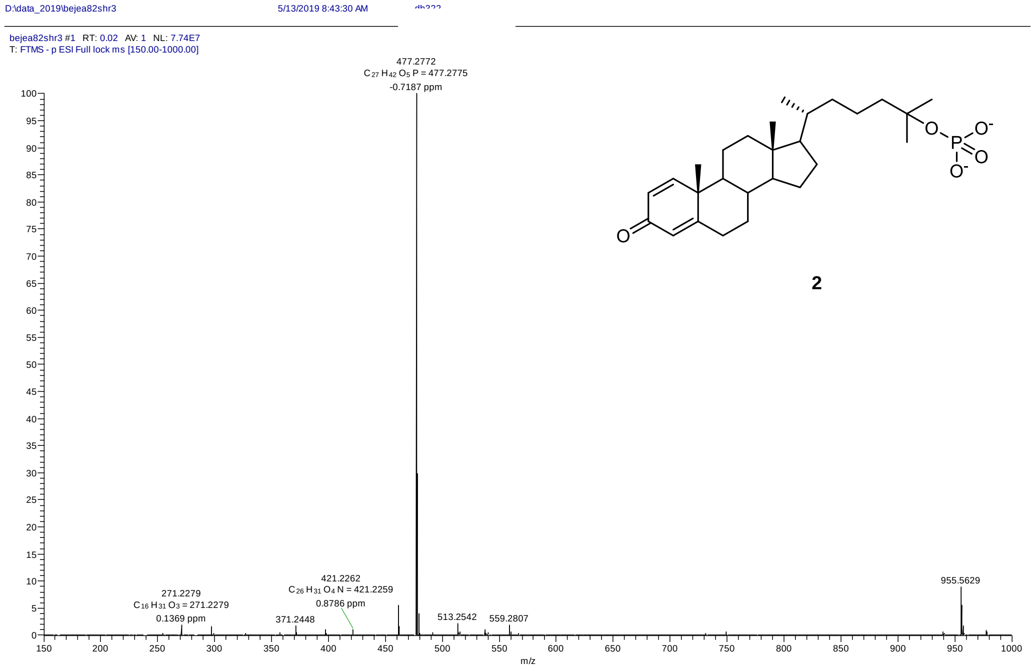

Supplementary Figure 5. (**a**) ^{31}P {^{1}H} NMR spectrum (162 MHz, MeOD-d$_4$) of chemically synthesized 25-phospho-CDO, (**2**) in **Fig. 2**. (**b**) ESI-Q-TOF MS spectrum of chemically synthesized 25-phospho-CDO, (**2**) in **Fig. 2**.

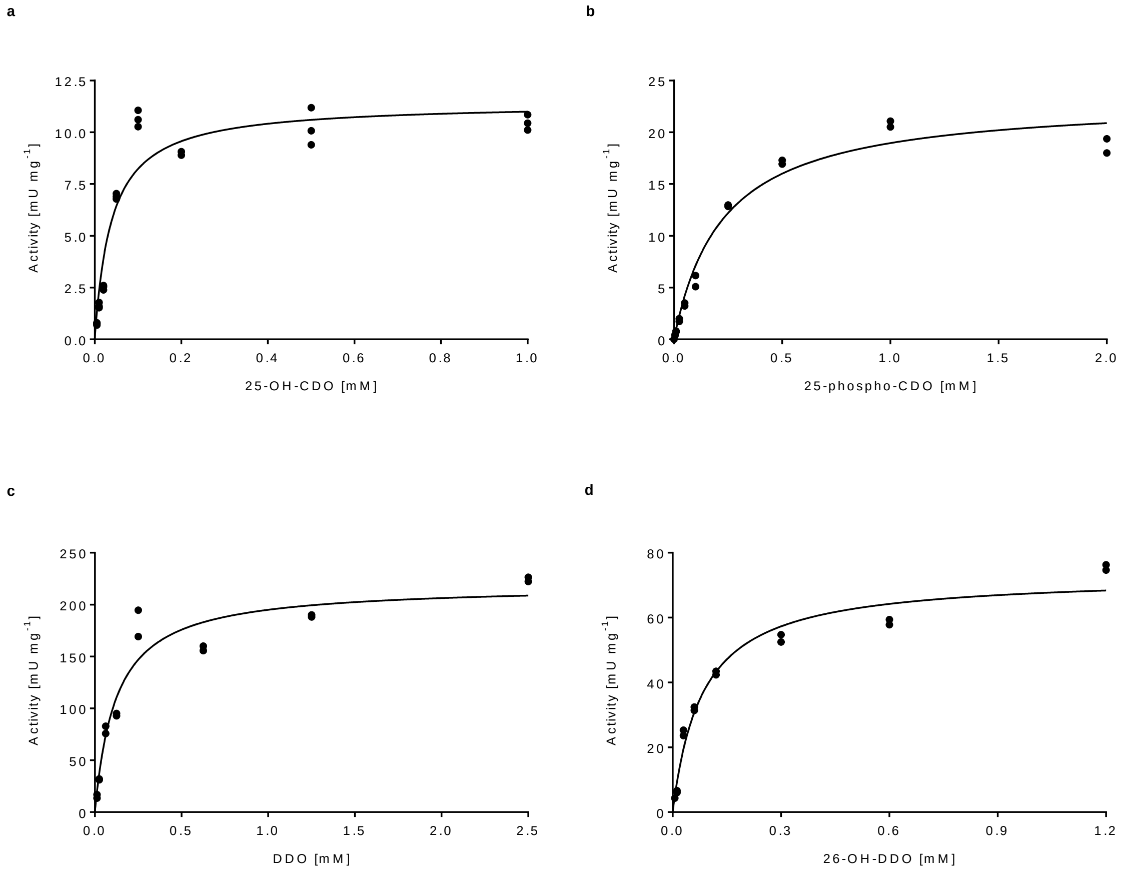

Supplementary Figure 6. Michaelis–Menten curves of enzymatic reactions analyzed. The data points shown were used to determine V_{max}- and K_m-values. Conversions by cell extracts from *S. denitrificans* enriched by 50% $(NH_4)_2SO_4$ precipitation: (**a**) ATP-dependent conversion of 25-OH-CDO to DDO. (**b**) ATP-independent conversion of 25-phospho-CDO to DDO. $K_3[Fe(CN)_6]$-dependent conversions by enriched S26DH. (**c**) hydroxylation of DDO to 26-OH-DDO. (**d**) Dehydrogenation of 26-OH-DDO to DDO-26-al. Source data are provided as a Source Data file.

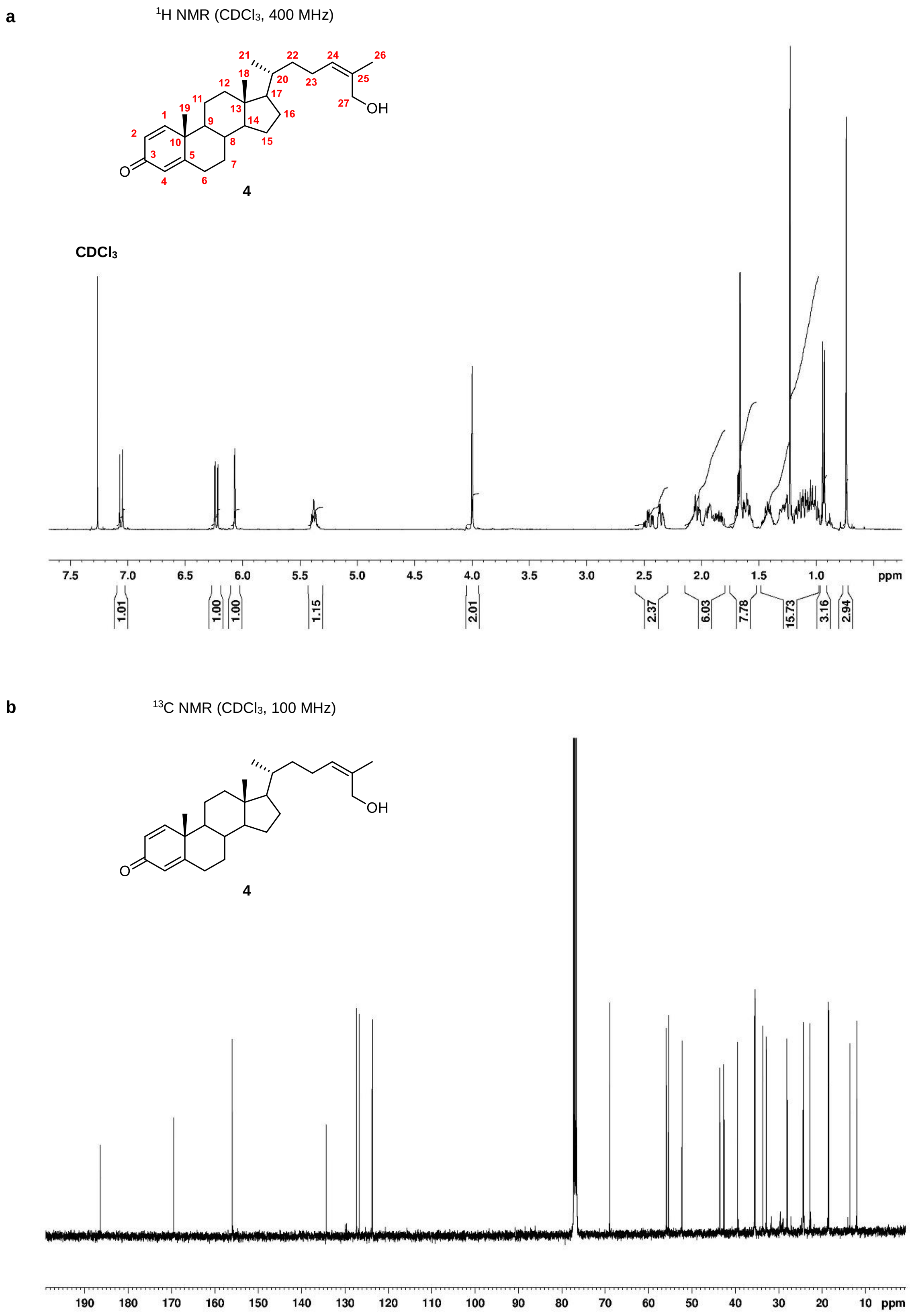

Supplementary Figure 7. ^{1}H (**a**) and ^{13}C (**b**) NMR spectra (400 MHz – ^{1}H, 100 MHz – ^{13}C, $CDCl_3$) of the product obtained during $K_3[Fe(CN)_6]$-dependent conversion of DDO by *S. denitrificans* cell extracts. The spectra are indicative for 26-OH-DDO, **4** in **Fig. 3** of the main text.

a

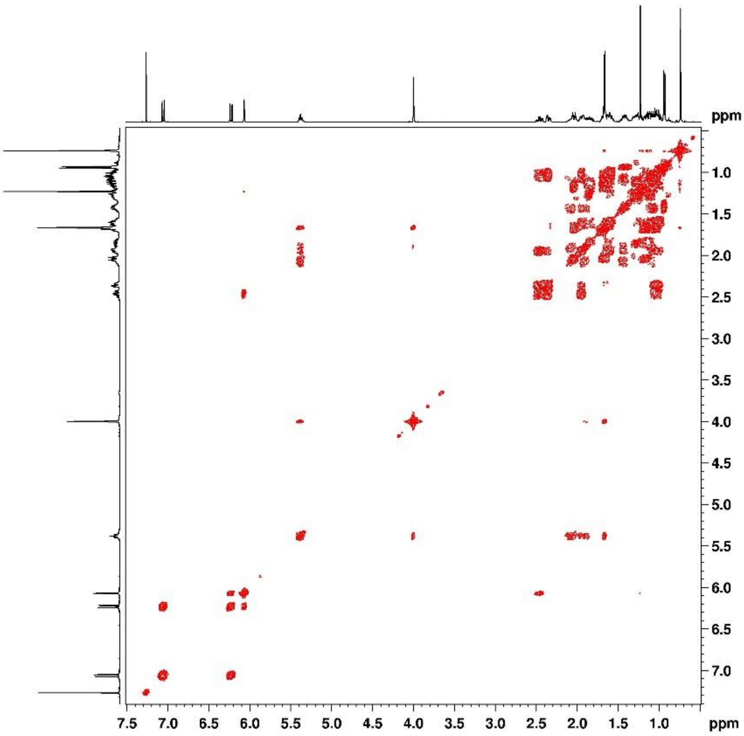

b

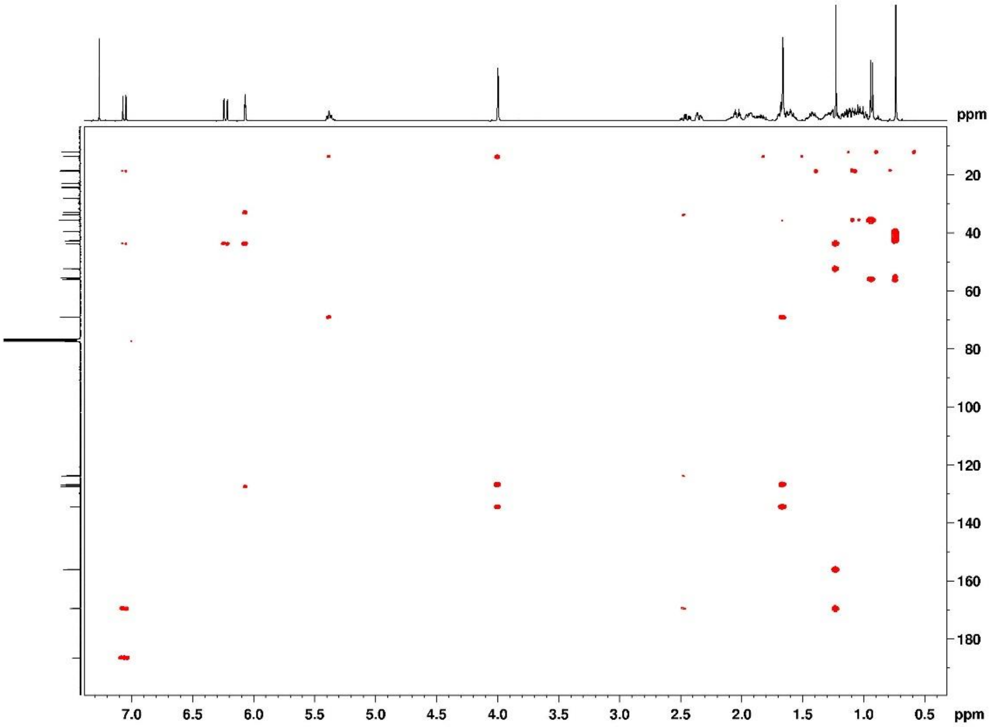

Supplementary Figure 8. HH COSY (**a**) and HMBC (**b**) NMR spectra of the product obtained during $K_3[Fe(CN)_6]$-dependent conversion of DDO by *S. denitrificans* cell extracts. The spectra are indicative for 26-OH-DDO, **4** in **Fig. 3** of the main text.

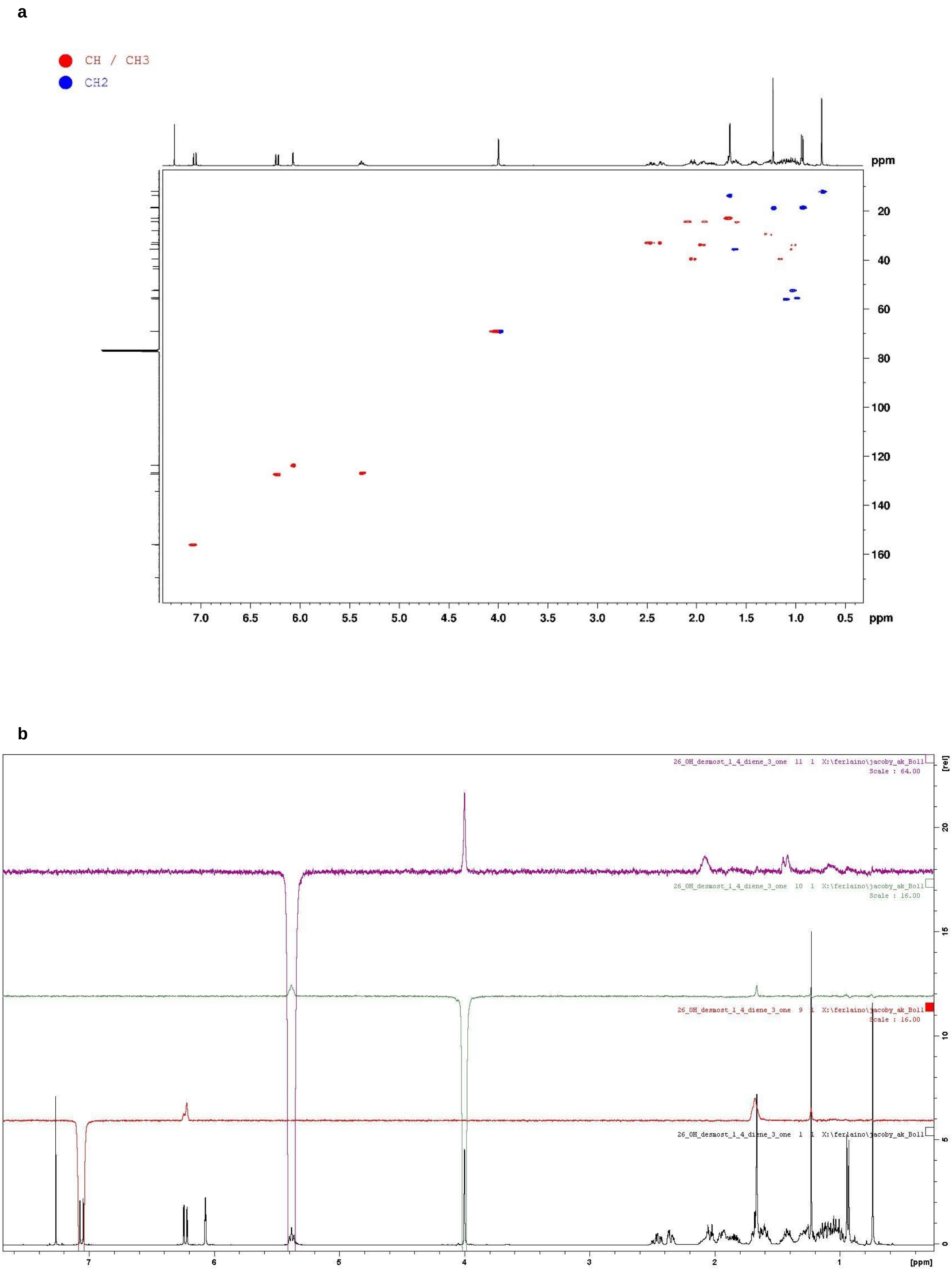

Supplementary Figure 9. HSQC (**a**) and NOESY (**b**) NMR spectra of the product obtained during $K_3[Fe(CN)_6]$-dependent conversion of DDO by *S. denitrificans* cell extracts. The spectra are indicative for 26-OH-DDO, **4** in **Fig. 3** of the main text.

a

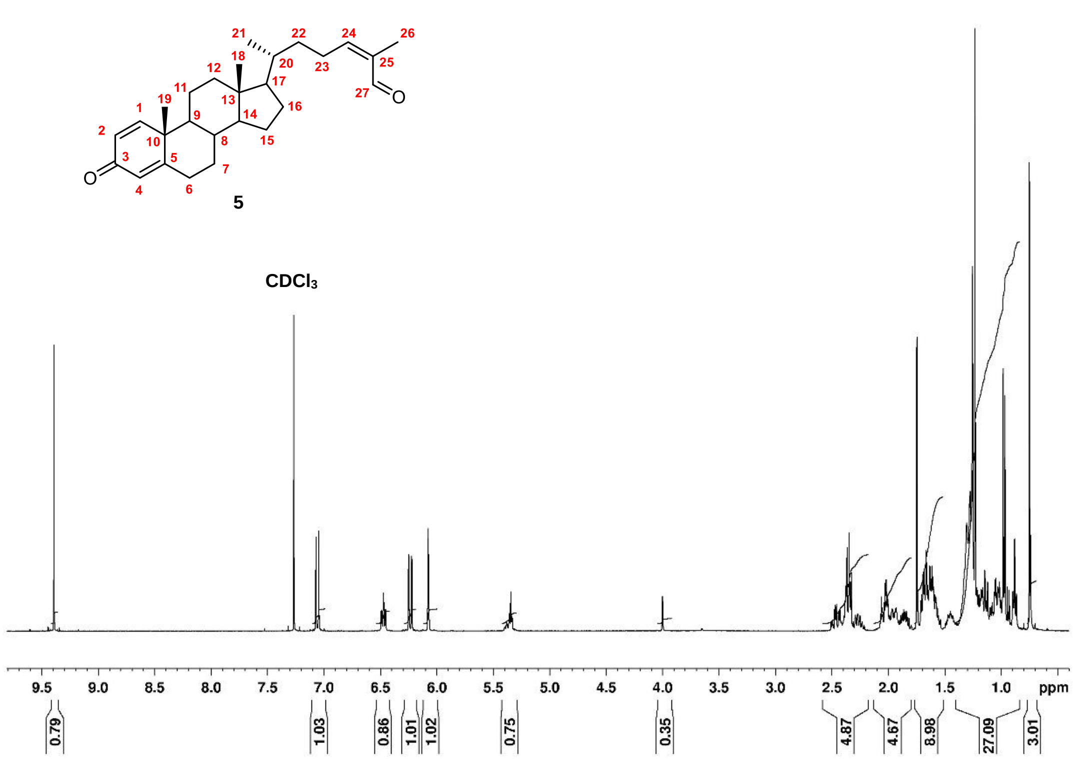

b

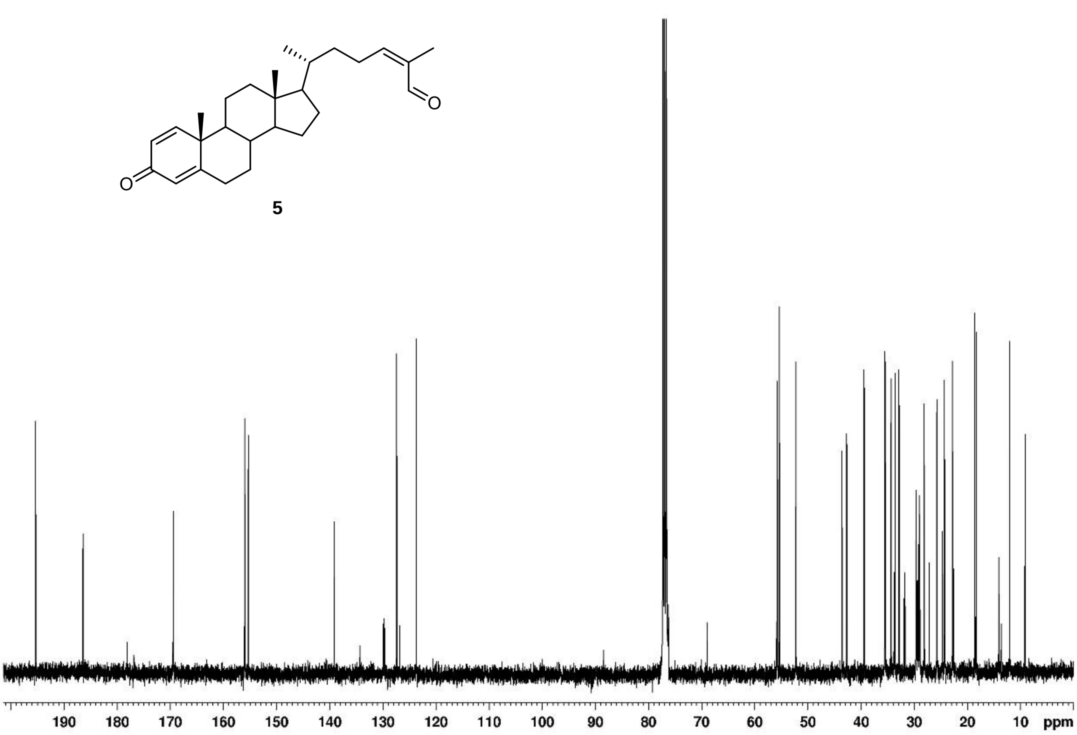

Supplementary Figure 10. ^{1}H (**a**) and ^{13}C (**b**) NMR spectra (400 MHz – ^{1}H, 100 MHz – ^{13}C, $CDCl_3$) of the product obtained during $K_3[Fe(CN)_6]$-dependent conversion of DDO by *S. denitrificans* cell extracts. The spectra are indicative for DDO-26-al, **5** in **Fig. 3** of the main text.

a

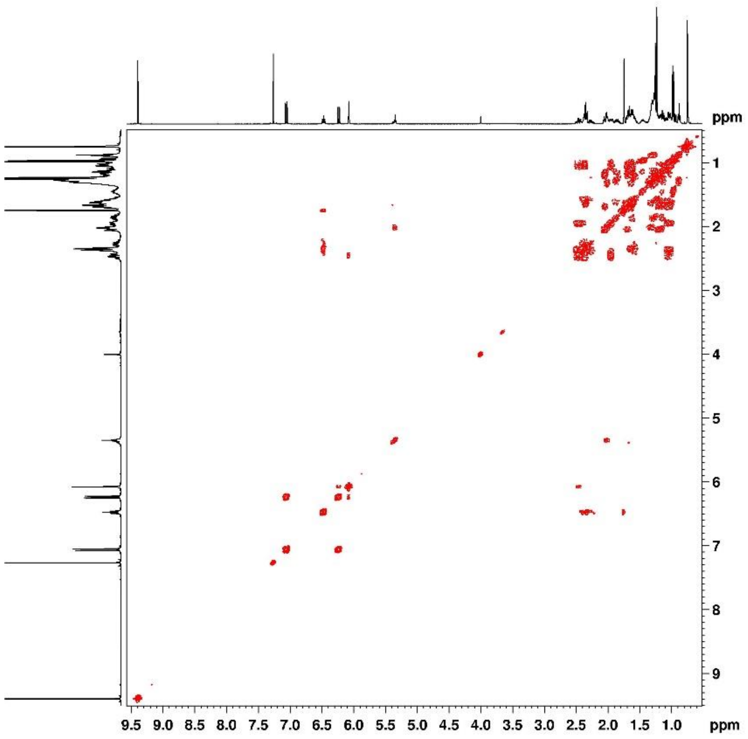

b

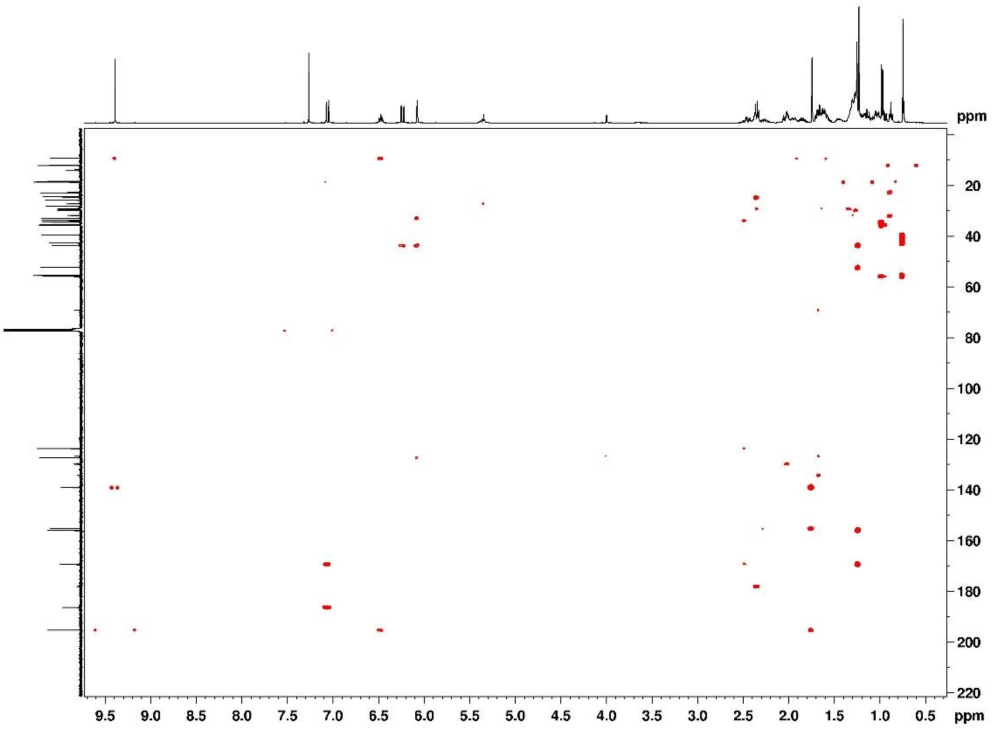

Supplementary Figure 11. HHCOSY (**a**) and HMBC (**b**) NMR spectra of the product obtained during $K_3[Fe(CN)_6]$-dependent conversion of DDO by *S. denitrificans* cell extracts. The spectra are indicative for DDO-26-al, **5** in **Fig. 3** of the main text.

a

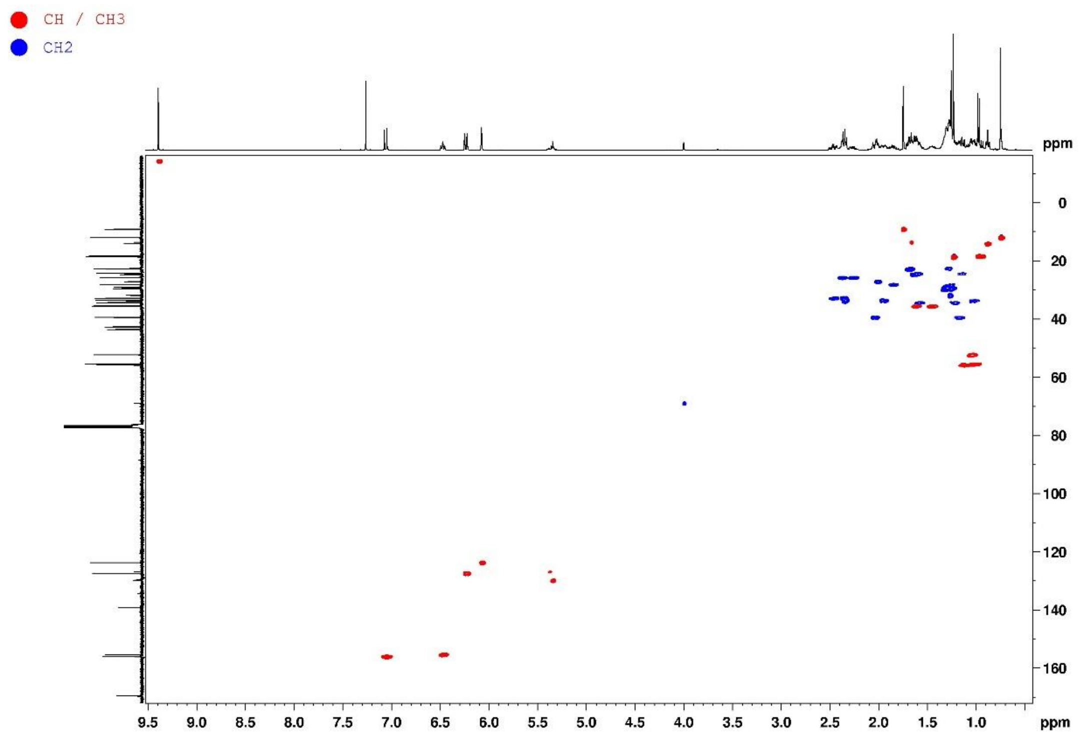

b

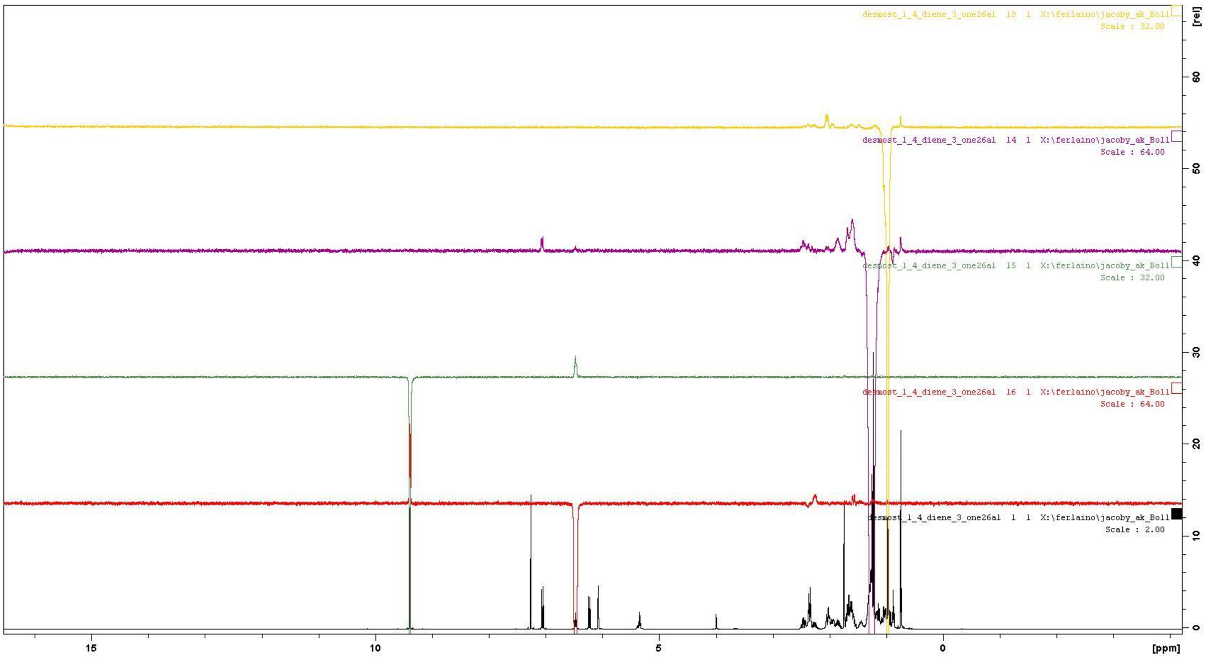

Supplementary Figure 12. HSQC (**a**) and NOESY (**b**) NMR spectra of the product obtained during $K_3[Fe(CN)_6]$-dependent conversion of DDO by *S. denitrificans* cell extracts. The spectra are indicative for DDO-26-al, **5** in **Fig. 3** of the main text.

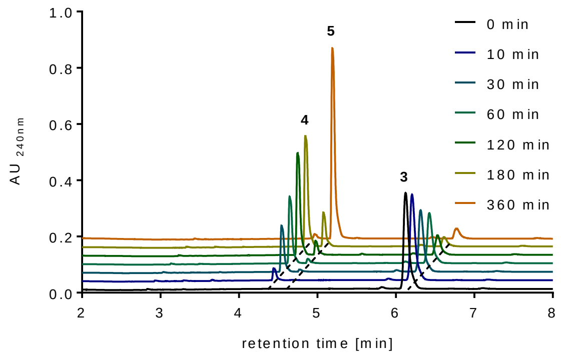

Supplementary Figure 13. Conversion of DDO with enriched S26DH$_1$. Time- and $K_3[Fe(CN)_6]$-dependent conversion of DDO **3** to the two products **4** and **5** (see **Figure 3** main text). Source data are provided as a Source Data file.

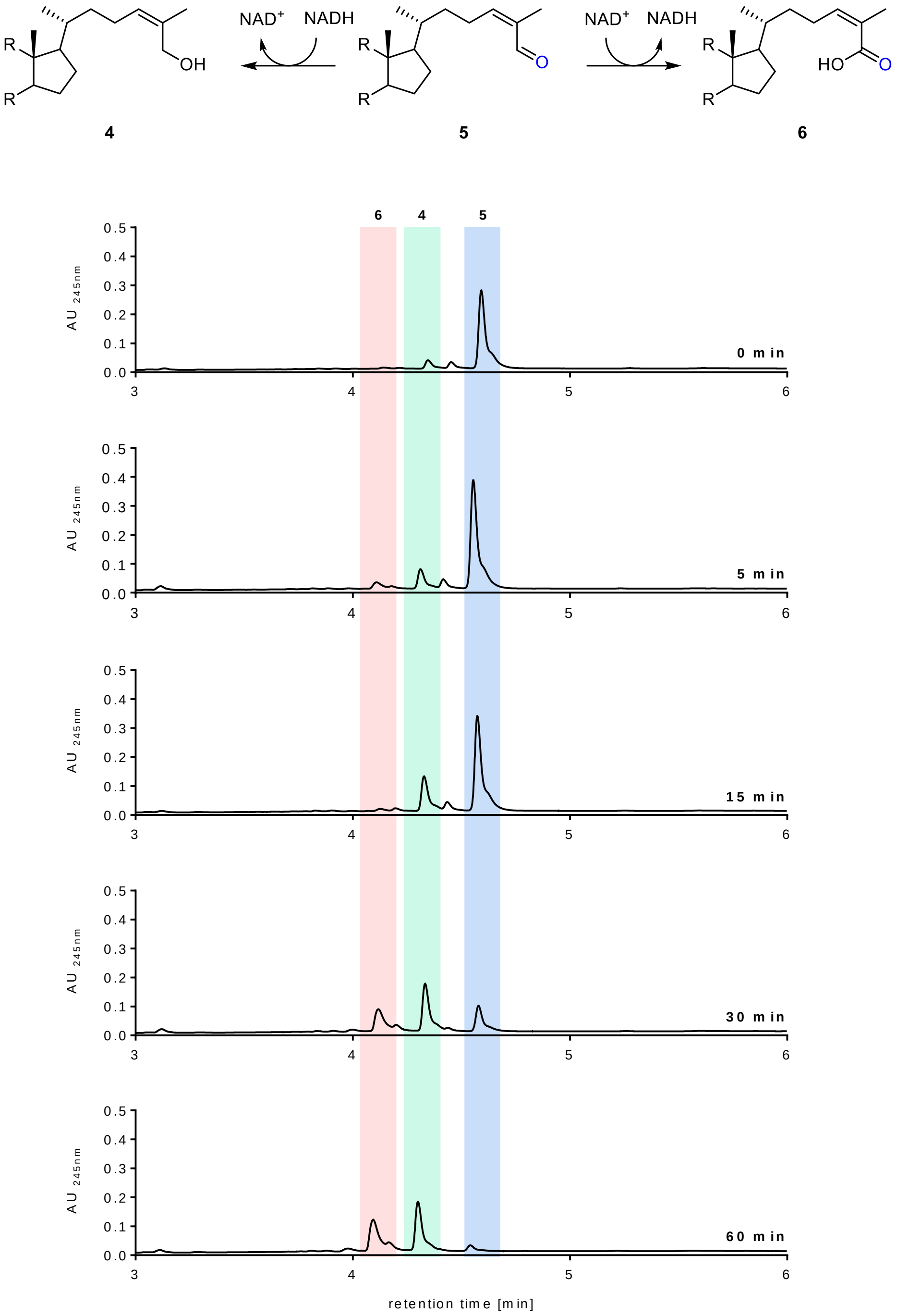

Supplementary Figure 14. NAD$^+$-dependent conversion of DDO-26-al (5) by cell extracts from *S. denitrificans*. DDO-26-al (**5**) was converted to DDO-26-oic acid (**6**). In parallel, reduction of DDO-26-al (**5**) to 26-OH-DDO (**4**) by an unspecific dehydrogenase using the NADH formed in the course of the reaction was observed. Assignment of peaks to structures is based on ESI-QTOF-MS analyses. Source data are provided as a Source Data file.

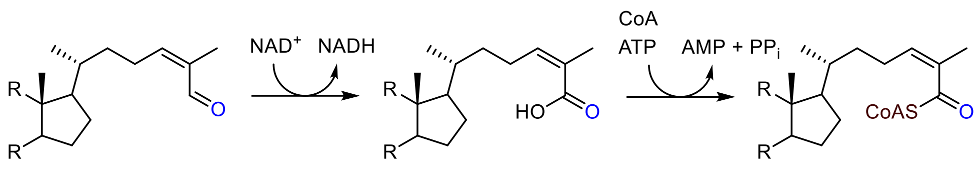

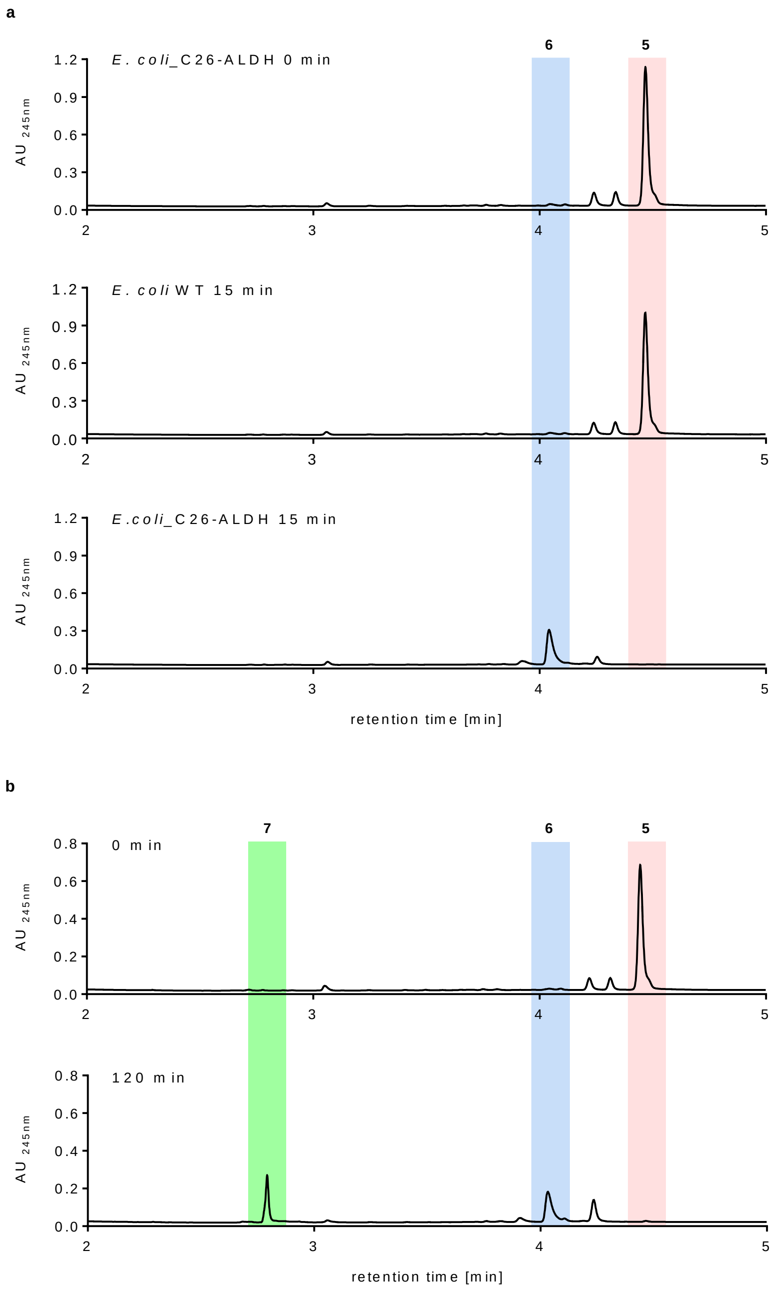

Supplementary Figure 15. Conversion of DDO-26-al to DDO-26-CoA. (**a**) NAD^+-dependent conversion of DDO-26-al (**5**) by cell extracts from heterologously produced DDO-26-al dehydrogenase (C26-ALDH) of *E. coli* BL21. (**b**) Conversion of DDO-26-al (**5**) to DDO-26-oic acid (**6**) and DDO-26-CoA (**7**) by cell extracts of *S. denitrificans* in the presence of MgATP, CoA and NAD^+. Source data are provided as a Source Data file.

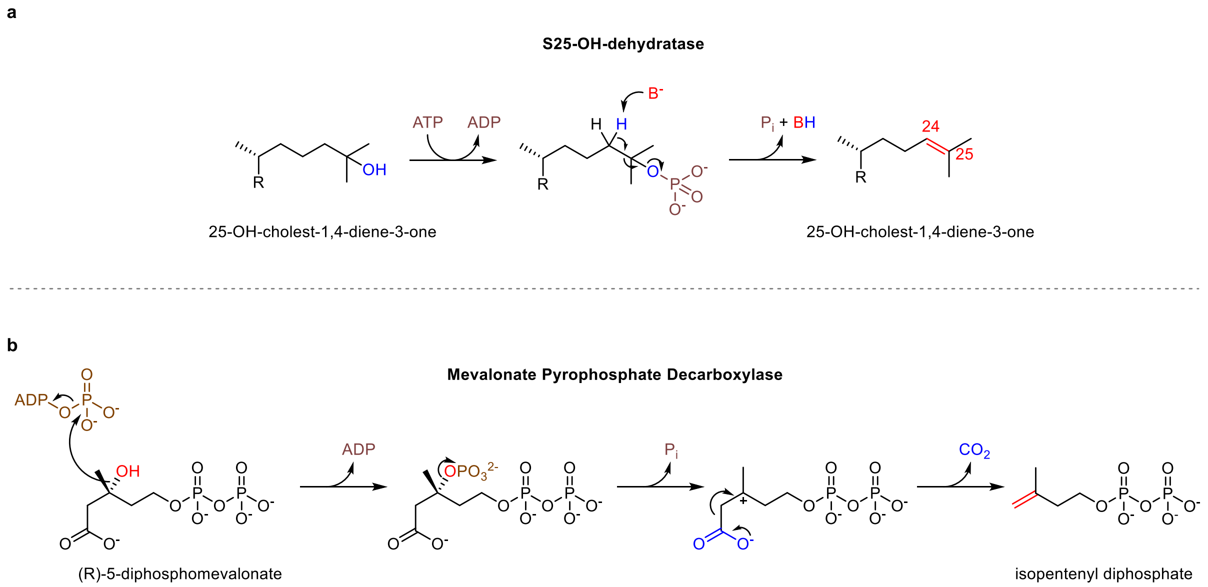

Supplementary Figure 16. ATP-dependent conversions of (**a**) S25-OH-dehydratase, and (**b**) mevalonate pyrophosphate decarboxylase.
