# Supplementary material for: ATP-dependent hydroxylation of an unactivated primary carbon with water
Source: Nat Commun. 2020 Aug 6;11:3906. doi: 10.1038/s41467-020-17675-7 (PMC7411048; doi:10.1038/s41467-020-17675-7)
Supplement: Supplementary file 3 — Reporting Summary [file 41467_2020_17675_MOESM3_ESM.pdf]

nature research

Corresponding author(s): Matthias Boll

Last updated by author(s): July 06 2020

Reporting Summary

Nature Research wishes to improve the reproducibility of the work that we publish. This form provides structure for consistency and transparency in reporting. For further information on Nature Research policies, see Authors & Referees and the Editorial Policy Checklist .

Statistics

For all statistical analyses, confirm that the following items are present in the figure legend, table legend, main text, or Methods section.

n/a	Confirmed	
☐	☒	The exact sample size (*n*) for each experimental group/condition, given as a discrete number and unit of measurement
☒	☐	A statement on whether measurements were taken from distinct samples or whether the same sample was measured repeatedly
☒	☐	The statistical test(s) used AND whether they are one- or two-sided<br>*Only common tests should be described solely by name; describe more complex techniques in the Methods section.*
☒	☐	A description of all covariates tested
☐	☒	A description of any assumptions or corrections, such as tests of normality and adjustment for multiple comparisons
☒	☐	A full description of the statistical parameters including central tendency (e.g. means) or other basic estimates (e.g. regression coefficient) AND variation (e.g. standard deviation) or associated estimates of uncertainty (e.g. confidence intervals)
☒	☐	For null hypothesis testing, the test statistic (e.g. *F*, *t*, *r*) with confidence intervals, effect sizes, degrees of freedom and *P* value noted<br>*Give P values as exact values whenever suitable.*
☒	☐	For Bayesian analysis, information on the choice of priors and Markov chain Monte Carlo settings
☒	☐	For hierarchical and complex designs, identification of the appropriate level for tests and full reporting of outcomes
☒	☐	Estimates of effect sizes (e.g. Cohen's *d*, Pearson's *r*), indicating how they were calculated

Our web collection on statistics for biologists contains articles on many of the points above.

Software and code

Policy information about availability of computer code

Data collection	There was no data collection with a software other than mentioned below under 'data analysis'.
Data analysis	GraphPad Prism 6, ProteinLynx Global Server 3.0.3, Mass Lynx 4.1, Topspin 3.2, Mnova, ImageLab 3.0.1.

For manuscripts utilizing custom algorithms or software that are central to the research but not yet described in published literature, software must be made available to editors/reviewers. We strongly encourage code deposition in a community repository (e.g. GitHub). See the Nature Research guidelines for submitting code & software for further information.

Data

Policy information about availability of data

All manuscripts must include a data availability statement. This statement should provide the following information, where applicable:

- Accession codes, unique identifiers, or web links for publicly available datasets
- A list of figures that have associated raw data
- A description of any restrictions on data availability

Data availability statement: The source data underlying Figs. 2ab, 3a, 4a, 4b, Supplementary Tables 1 and 2, Supplementary Figs. 4a-d, 7, 8, and 9ab are provided in the Source Data file. Additional data that support the findings of this study are available from the corresponding author upon reasonable request. .

Field-specific reporting

Please select the one below that is the best fit for your research. If you are not sure, read the appropriate sections before making your selection.

☒ Life sciences ☐ Behavioural & social sciences ☐ Ecological, evolutionary & environmental sciences

Life sciences study design

All studies must disclose on these points even when the disclosure is negative.

Sample size	Sample sizes for determination of kinetic parameters and metal content are indicated in the text, original data are shown in the source file.
Data exclusions	No data were excluded
Replication	Mean values +/- standard deviations for determination of kinetic parametersare indicated and were in most cases 3 biological replicates
Randomization	For the quantitative biochemical determination of enzyme activities, kinetic parameters enzyme cofactor content, randomization is not relevant. Our enzyme characterization did not depend on sophisticated statistical analyses of an unknown relationship, rather than on the specific characterization of the properties of a defined enzyme. Stastics were limited to determination of mean values +/- SD deviations which is usual for such initial analyses.
Blinding	For the quantitative biochemical determination of enzyme activities, kinetic parameters enzyme cofactor content, blinding is not relevant. Elucidating of a defined enzyme cascade for adefined pathway requires specific enzymatic assays where blinding is not applicable.

Reporting for specific materials, systems and methods

We require information from authors about some types of materials, experimental systems and methods used in many studies. Here, indicate whether each material, system or method listed is relevant to your study. If you are not sure if a list item applies to your research, read the appropriate section before selecting a response.

Materials & experimental systems

n/a	Involved in the study	
☒	☐	Antibodies
☒	☐	Eukaryotic cell lines
☒	☐	Palaeontology
☒	☐	Animals and other organisms
☒	☐	Human research participants
☒	☐	Clinical data

Methods

n/a	Involved in the study	
☒	☐	ChIP-seq
☒	☐	Flow cytometry
☒	☐	MRI-based neuroimaging
